# The effect of simvastatin treatment on endothelial cell response to shear stress and tumor necrosis factor alpha stimulation

**DOI:** 10.1186/s12938-015-0057-x

**Published:** 2015-06-20

**Authors:** Melissa Dick, Katherine MacDonald, Jean-Claude Tardif, Richard L Leask

**Affiliations:** Department of Chemical Engineering, McGill University, 3610 University Avenue, Montreal, QC H3A 2B2 Canada; Montreal Heart Institute, 5000 Belanger Street, Montreal, QC H1T 1C8 Canada

**Keywords:** Endothelial cells, Morphology, Shape index, Three dimensional tissue culture, Actin cytoskeleton, Mechanotransduction, Statins, Tumor necrosis factor alpha

## Abstract

**Background:**

Statin drugs are one of the most commonly prescribed pharmaceuticals by physicians. By blocking the rate-limiting step in the cholesterol biosynthesis pathway, statins inhibit cholesterol synthesis, which benefits patient health. However, since many other important cellular processes are regulated within this pathway, they may also be influenced by statin therapy. These pleiotropic effects of statins have not been fully investigated, but are believed to positively influence endothelial cells (ECs), which line the vasculature in a confluent monolayer. Few studies have considered the effect of blood flow on ECs and how this may augment EC response to statins.

**Methods:**

In this study, the effect of statin treatment on ECs is investigated for cells stimulated with tumor necrosis factor alpha (TNF-α), an inflammatory cytokine that promotes an atheroprone endothelium. Additionally, ECs are exposed to a physiologically relevant wall shear stress (WSS) of 12.5 dynes/cm^2^ using a three-dimensional tissue culture model to provide a realistic hemodynamic environment. ECs are analyzed for morphology using light microscopy as well as cytoskeletal structure and alignment using confocal microscopy. Statistical analysis is performed on the results using both the one-way analysis of variance with Bonferroni post-tests and the two-tailed *t* test.

**Results:**

We have shown that statin treatment caused cells to adapt to a rounded, atheroprone morphology, with a significantly higher shape index. Oppositely, TNF-α stimulation caused cells to elongate to an atheroprotective morphology, with a significantly lower shape index. WSS and TNF-α were unable to reverse any statin-induced cell rounding or F-actin disruption.

**Conclusion:**

Further work is therefore needed to determine why statin drugs cause cells to have an atheroprone morphology, but an atheroprotective genotype, and why TNF-α stimulation causes an atheroprotective morphology, but an atheroprone genotype. Despite the morphological changes due to statins or stimulation, ECs still respond to WSS. Understanding how statins influence ECs will allow for more targeted treatments for hypercholestemia and potentially other diseases.

## Introduction

Despite improved treatment options available, cardiovascular diseases, such as atherosclerosis, remain a leading cause of death in North America [1]. Atherosclerosis is the accumulation of cells, lipids, and extracellular matrix within blood vessel walls that can lead to the formation of an unstable plaque, causing heart attack or stroke [2–4]. High cholesterol levels are one of many risk factors for developing atherosclerosis [5].

Clinically, statin drugs [inhibitors of the enzyme 3-hydroxy-3-methylglutaryl coenzyme A (HMG-CoA) reductase] have been successful in reducing patient cholesterol levels [6–11], reducing the risk of heart attack and stroke. There are other benefits to statin therapy that the decrease in cholesterol alone cannot explain, called pleiotropic effects [10, 12–14]. These effects are believed to be regulated in side pathways of the cholesterol biosynthesis pathway [10, 15], which is responsible for cellular processes such as cellular movement, signaling, morphology, differentiation, and cytoskeletal remodeling [10, 16, 17]. Statin drugs are thought to positively influence endothelial cells (ECs), the cells that line the vasculature in a confluent monolayer [2, 12–14].

ECs are a dynamic interface between the blood and vascular tissue and play a pivotal role in disease formation. Dysfunction of the ECs in response to biochemical and hemodynamic stresses can create an inflammatory phenotype associated with atherosclerosis [18, 19]. Early work on the pleiotropic effects of statins neglected the effect of these environmental stimuli and instead focused on static and in vivo studies, reviewed by Liao and Laufs [20] and Sadowitz et al. [21]. Recent work has shown that this response is altered in the presence of fluid wall shear stress (WSS) [22–25].

EC morphology is believed to be a key indicator of cellular health [17, 26, 27]. Elongated ECs are believed to be healthy, as they are found in regions of the vasculature exposed to undisturbed flow and are less likely to become atherogenic [18, 28]. Oppositely, rounded ECs are thought to be unhealthy as they are found in regions of disturbed blood flow, such as around curvature or bifurcations, where atherosclerotic lesions are likely to occur [2, 18, 28–31]. Both EC morphology and F-actin cytoskeleton structure are regulated within the cholesterol biosynthesis pathway [32, 33], and may therefore be influenced by statin treatment. Indeed, recent work published by our lab showed that ECs treated with statin drugs became rounded with a fragmented F-actin cytoskeleton when compared to an untreated control [25]. This process may be influenced by the activity of the small GTPase RhoA [28, 34], regulated within the cholesterol biosynthesis pathway [10, 15].

As atherosclerosis is an inflammatory disease, we have continued our studies by looking at the response of an inflamed endothelium to statin therapy. Tumor necrosis factor alpha (TNF-α) is implicated in various inflammatory diseases and is commonly used to simulate an inflamed, atheroprone EC phenotype [35, 36]. TNF-α is also believed to influence the EC cytoskeleton through RhoA activation [37]. Here, we describe how EC morphology and F-actin cytoskeletal arrangement is affected when TNF-α stimulated cells are treated with simvastatin, under static and flow conditions.

## Methods

The methods used here are based upon methods previously described elsewhere [22–25], but will be summarized briefly.

### Tissue culture model preparation

Flexible, transparent tissue culture models were prepared by curing Sylgard^®^ 184 silicone elastomer (Dow Corning) around a 2 mm diameter stainless steel rod (McMaster-Carr, 1256T999) in a Plexiglas mold for 24 h at 45°C. To sterilize for cell culture, models were boiled in reverse osmosis water for 30 min. A suitable surface for cell growth was created by coating the inner lumen of the model with 40 μg/mL fibronectin (Sigma, F0985). The models were sealed using PVDF filters (Fisher, 097203) and attached to a LabQuake rotor (Barnstead/Thermolyne) and left to rotate overnight within an incubator set at 37°C and 5% CO_2_ (Thermo Scientific).

### Cell culture

Human abdominal aortic endothelial cells (HAAECs) from a 20-year-old male (Coriell Institute for Medical Research, AG09799) were obtained from the supplier and expanded as per supplier protocol. For individual experiments, 1 mL of frozen HAAECs was thawed and cultured within a T175 flask (VWR, 82050-872) coated with gelatin (Sigma, G2500) using basal PromoCell media with SupplementMix (PromoCell, C-22010) containing 0.02 mL/mL fetal calf serum, 0.004 mL/mL endothelial cell growth supplement, 0.1 ng/mL human recombinant epidermal growth factor, 1 ng/mL human recombinant basic fibroblast growth factor, 90 μg/mL heparin, and 1 μg/mL hydrocortisone, with 10% fetal bovine serum (Invitrogen, 26140-079) and 1% penicillin–streptomycin (Invitrogen, 15140-122). The media was changed every 48 h and HAAECs were split into 4 T175 flasks after 4 days when they became confluent. HAAECs were seeded into the fibronectin-coated models at passage 5, with a concentration of 1.25 × 10^6^ cells/mL, and left on the rotor for 24 h to create a confluent monolayer of cells within the tissue culture models.

### Solution preparation

Simvastatin (Sigma, S6196), mevalonate (Sigma, M4667), and TNF-α (Cedarlane Laboratories, 300-01A) were obtained from the suppliers and stock solutions of 0.5 mM simvastatin, 50 mM of mevalonate, and 0.002 mg/mL TNF-α were prepared. These stock solutions were added to either the perfusion media, or within the lumen of static models, to obtain the final working concentrations of 10 μM simvastatin, 200 μM mevalonate, and 10 ng/mL TNF-α. MTT assays were used to ensure no significant cell death due to simvastatin, mevalonate, or TNF-α treatment (unpublished data). These particular concentrations were chosen to elicit a reproducible response in vitro [24], although they are higher than a typical clinical dose [38].

### Perfusion experiment

**Figure 1:**
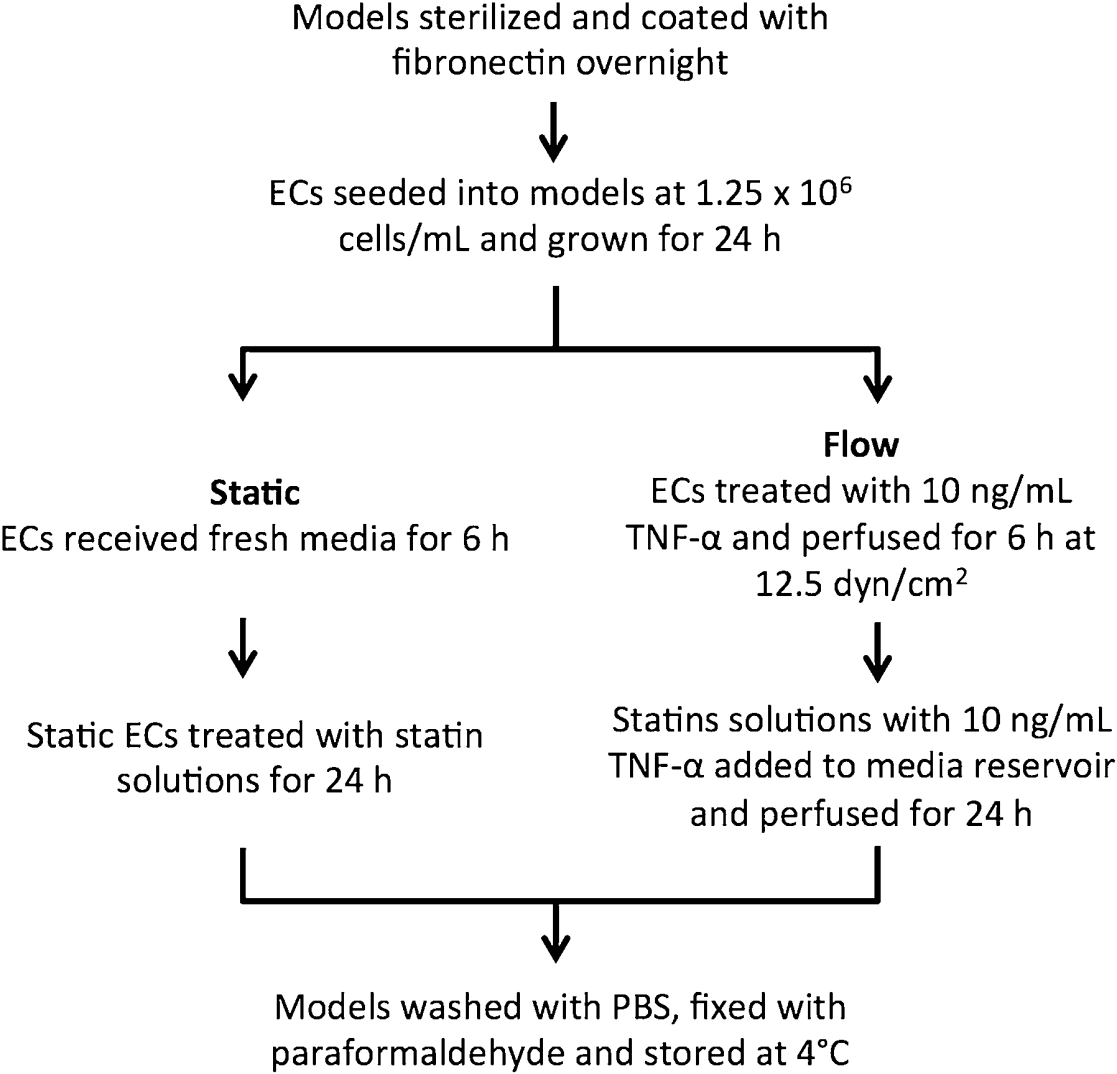
Experimental timeline detailing the endothelial cell model preparation, perfusion experiment set up, and statin treatment.

A flow diagram of the experimental time line is shown in Figure 1. A flow loop was created within an incubator to ensure a proper environment for the cells was available for the duration of the experiment. The flow loop consisted of tubing (Cole Parmer), a peristaltic pump (Ismatec), custom stainless steel flow dampeners, and a media reservoir with a multi-hole cap. Both static and perfused models were analyzed. 6 h of preshearing was used in these experiments to first elongate the perfused cells to a morphology consistent with those found within the blood vessel of a patient starting statin treatment. For static models, the cells were not sheared for 6 h, but were instead kept static with fresh media.

All perfused models were sheared at 12.5 dynes/cm^2^, a hemodynamically relevant level of WSS [29]. At t = 0 h, perfused models were introduced into the flow loop, and static models received new media. For perfused models being treated with TNF-α, 10 ng/mL TNF-α was introduced at this time. Static models had no TNF-α addition at this time. At t = 6 h, static models had a media change to introduce the statin solutions. For the perfused models, statin solutions, with or without 10 ng/mL TNF-α, were added directly to the perfusion media. A control with no statins was run in parallel, with or without 10 ng/mL TNF-α. The experiment was continued for a further 24 h of treatment time. At t = 30 h, the models were disconnected from the flow loop. All models were washed three times with 1× PBS, fixed with 1% paraformaldehyde (Sigma-Aldrich, P6148) for 20 min, and stored at 4°C until analysis.

### Tissue culture model analysis

Models were analyzed for both EC morphology and F-actin cytoskeleton structure. For morphology analysis, the models were cut into 1 cm sections and then cut in half through the lumen. The bottom half of the model was analyzed by staining the EC nucleus with a 4% solution of crystal violet (BD Biosciences, 212525) in 1× PBS for 9 min and viewed under a light microscope (Leica Microsystems, DMIL Microscope) with digital camera (Leica Microsystems, Leica DC300). A single image was taken of each model piece, with ten representative ECs analyzed from each image. Images were analyzed using Photoshop (Adobe) and a MATLAB^®^ (Mathworks) code to analyze the shape index (SI), a metric that ranges from 0 to 1 [39–42], describing how rounded the cell is; the closer the SI is to 1, the rounder the cell. The code recognizes the boundaries of the chosen representative cell based upon pixel intensity to determine the area, perimeter, and shape index of the EC nucleus, routinely used to quantify cell morphology [25, 39, 43].

To analyze the F-actin cytoskeleton, 1 cm model sections were cut and the ECs were blocked and permeabilized with 0.1% Triton™ X-100 (Sigma, T8787) at room temperature for 5 min. ECs were washed and then dyed with phalloidin-conjugated Alexa Fluor^®^ 647 (Invitrogen, A22287), at a 1:40 dilution in 1× PBS with 1% bovine serum albumin (Sigma-Aldrich, A7906) at room temperature. ECs were rinsed and the model was further cut so that only the bottom of the channel remained. The model piece was mounted within a cover slip bottomed petri dish (MatTek, CSGK) using a 0.2% Dabco (Sigma, D2522) solution in glycerol. A laser scanning confocal microscope (Zeiss, LSM 510) with Zen (2008) software (Zeiss) was used to image the ECs with a 10×/0.3 DICI or 20×/0.5 DICII objective lens. Image analysis was completed with Photoshop (Adobe) and ImageJ (National Institutes of Health).

### Statistical analysis

Each experiment was performed independently in triplicate. Prism™ 5 (GraphPad) was used to analyze the data. One-way analysis of variance (one-way ANOVA) with Bonferroni post-tests or unpaired two-tailed t tests were used. A P value of less than 0.05 was considered to be statistically significant.

## Results

### TNF-α elongates ECs under static conditions

**Figure 2:**
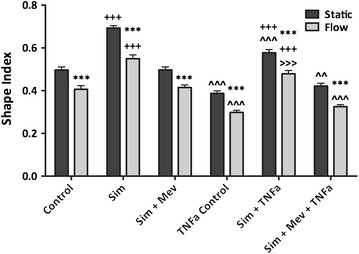
For each condition considered, the addition of 12.5 dynes/cm^2^ of steady wall shear stress caused a significant decrease in the endothelial cell shape index when compared to static ECs, ***P < 0.001 (two-tailed t test). The addition of 10 μM simvastatin caused a significant increase in the endothelial cell shape index for both non-stimulated and TNF-α stimulated endothelial cells, for both static and flow conditions when compared to the vehicle control condition, ^+++^P < 0.001 (one-way analysis of variance with Bonferroni post-tests). Stimulation with 10 ng/mL TNF-α caused a significant decrease in the endothelial cell shape index when compared to non-stimulated endothelial cells for all conditions considered, and for both static and flow conditions, ^^^^^P < 0.001, ^^^^P < 0.01 (one-way analysis of variance with Bonferroni post-tests),^>>>^P < 0.001 (two-tailed t test). For Sim + TNFa: N = 9; all other conditions: N = 10. These values are reported as the mean ± standard error. *Control* vehicle control, *Sim* simvastatin, *Mev* mevalonate, *TNFa* TNF-α.

**Figure 3:**
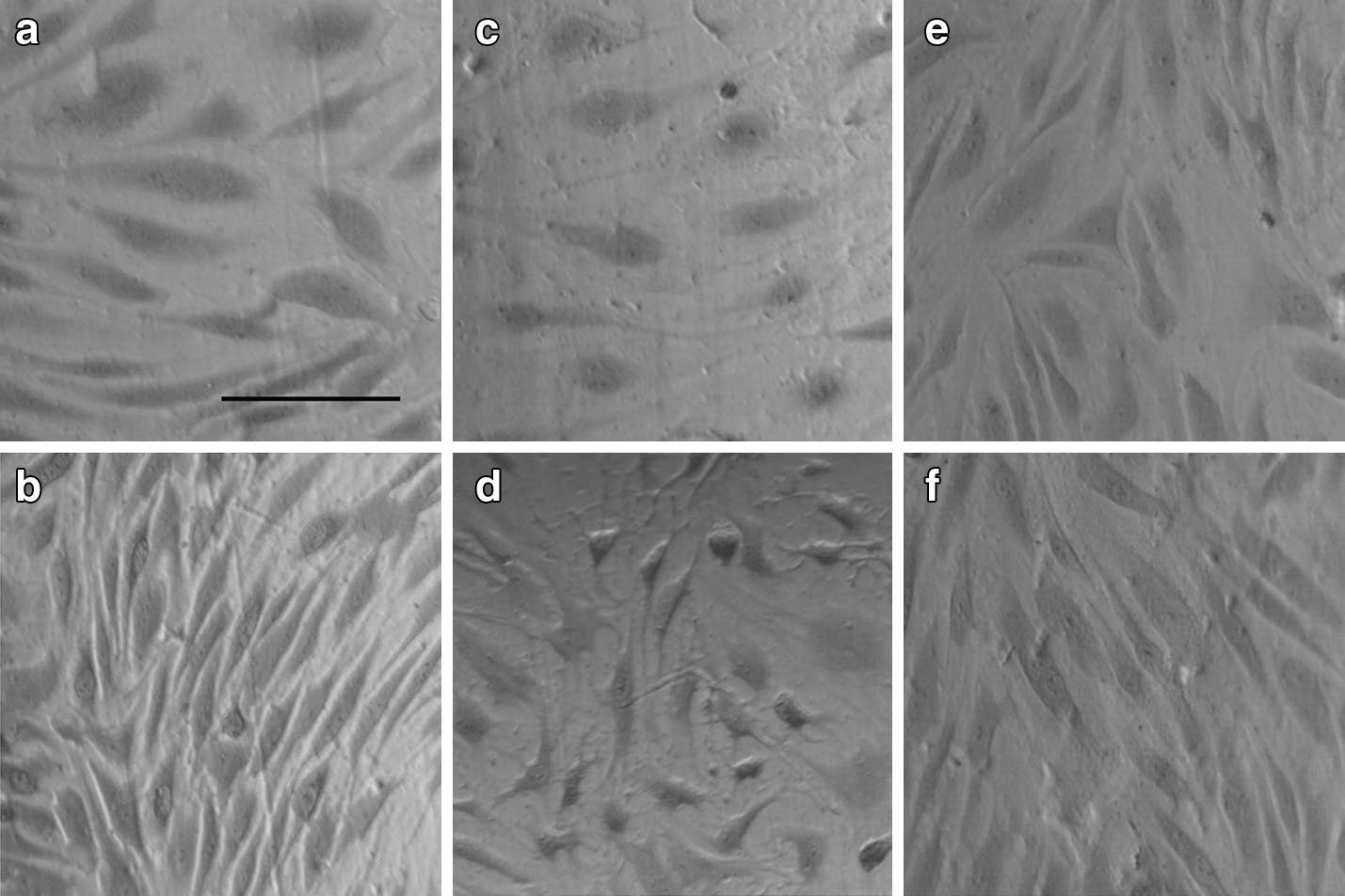
Endothelial cells stained with 4% crystal violet, a nuclear stain, and imaged at ×10 magnification. *Scale bar* represents 100 μm. All images are of endothelial cells that have been stimulated with 10 ng/mL TNF-α. Flow is in the *vertical direction*. **a** static, control; **b** flow, control; **c** static, simvastatin; **d** flow, simvastatin; **e** static, simvastatin and mevalonate; **f** flow, simvastatin and mevalonate.

Stimulation with 10 ng/mL TNF-α for 24 h caused a significant decrease in the SI when compared to the non-stimulated, static control, Figure 2 (P < 0.001, one-way ANOVA with Bonferroni post-tests). Visually, ECs appeared more elongated, Figure 3a.

### TNF-α stimulated ECs respond to WSS and elongate

When ECs were stimulated with 10 ng/mL TNF-α at a WSS of 12.5 dynes/cm^2^, there was a significant decrease in SI when compared to the non-stimulated, perfused control, Figure 2 (P < 0.001, one-way ANOVA with Bonferroni post-tests). Visually, ECs appeared more elongated, Figure 3b.

### Statin drugs round TNF-α stimulated ECs under static conditions

A significant increase in the SI was observed when TNF-α stimulated ECs were treated with 10 μM simvastatin under static conditions when compared to the TNF-α stimulated, static control cells, Figure 2 (P < 0.0001, unpaired two-tailed t test). Visually, ECs appeared more rounded, Figure 3c.

### Statin treated and TNF-α stimulated ECs respond to WSS and become rounded

Stimulated ECs exposed to 12.5 dynes/cm^2^ of WSS and treated with 10 μM simvastatin had a significantly higher SI than the TNF-α stimulated, perfused, control cells, Figure 2 (P < 0.001, one-way ANOVA with Bonferroni post-tests). Visually, ECs appeared more rounded, Figure 3d.

### Steady WSS and TNF-α stimulation have similar impacts on EC SI

When non-stimulated ECs exposed to flow and TNF-α stimulated ECs left under static conditions were compared, there was no significant difference observed, Figure 2 (P > 0.05, one-way ANOVA with Bonferroni post-tests).

### Both TNF-α stimulation and steady WSS are required to abrogate all statin effects on EC SI

There is no significant difference in EC SI when the TNF-α stimulated ECs were treated with 10 μM simvastatin and exposed to 12.5 dynes/cm^2^ of WSS when compared to the static control, Figure 2 (P > 0.05, unpaired two-tailed t test).

### TNF-α stimulation does not abrogate statin-induced F-actin cytoskeleton disorganization

**Figure 4:**
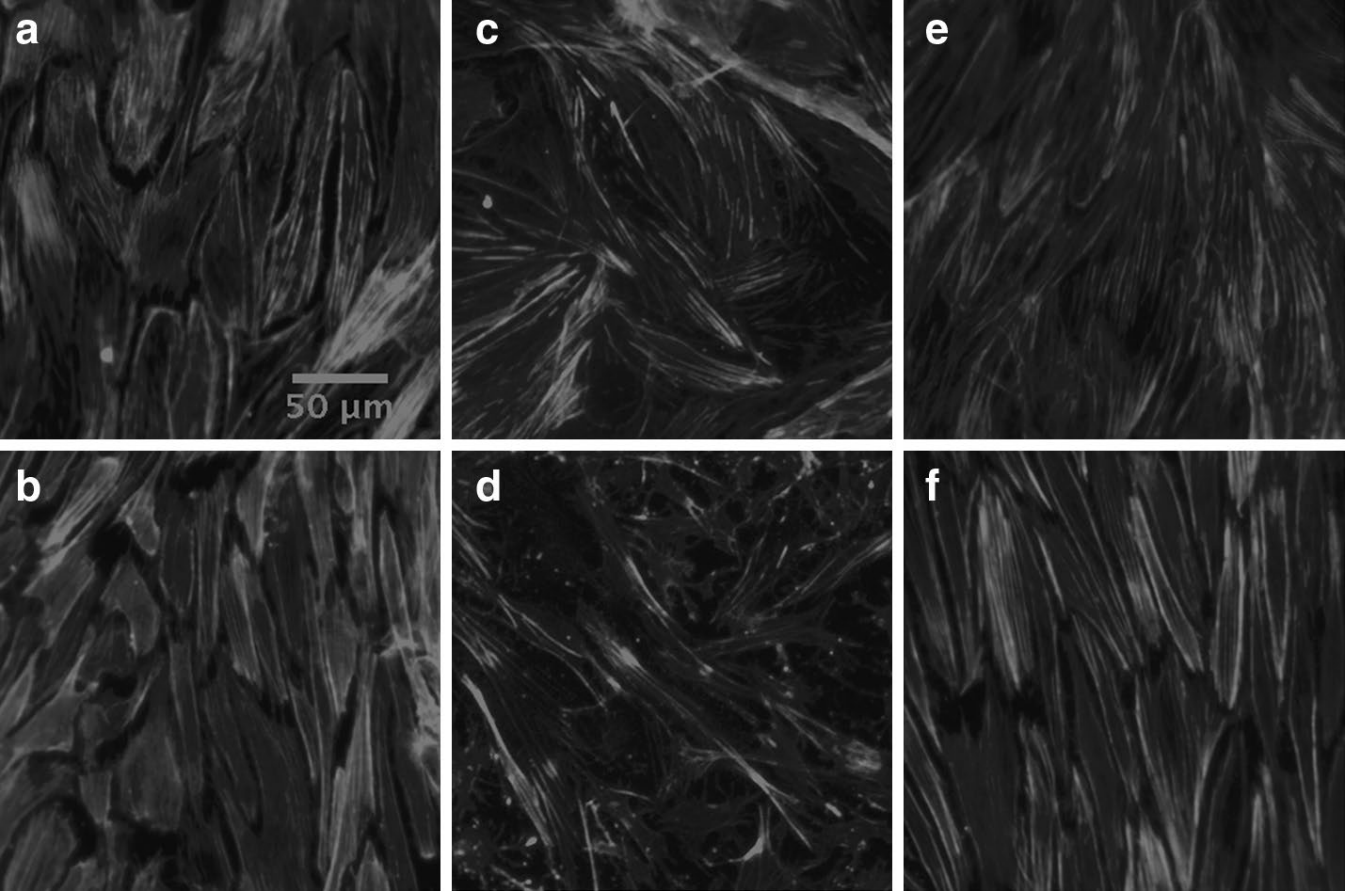
Endothelial cell F-actin cytoskeleton stained with phalloidin conjugated Alexa Fluor^®^ 647 and imaged using confocal microscopy. *Scale bars* represent 50 μm. All images are of endothelial cells that have been stimulated with 10 ng/mL TNF-α. Flow is in the *vertical direction*. **a** static, control; **b** flow, control; **c** static, simvastatin; **d** flow, simvastatin; **e** static, simvastatin and mevalonate; **f** flow, simvastatin and mevalonate.

When TNF-α stimulated ECs were treated with 10 μM simvastatin, the F-actin cytoskeleton became fragmented and disorganized. This was observed for both static and flow conditions, Figure 4c and d.

### Mevalonate abrogates the morphological change due to statin treatment of TNF-α stimulated ECs under static and flow conditions

When TNF-α stimulated ECs were treated with both 10 μM simvastatin and 200 μM mevalonate there was no significant difference from the TNF-α stimulated control cells, under both static and perfused conditions, Figure 2 (one-way ANOVA with Bonferroni post-tests). Visually, ECs appeared to have the same morphology as the control cells, Figure 3e and f.

### Mevalonate abrogates the change in F-actin cytoskeletal arrangement due to statin treatment of TNF-α stimulated ECs under static and flow conditions

When TNF-α stimulated ECs were treated with both 10 μM simvastatin and 200 μM mevalonate, the statin-induced F-actin cytoskeleton fragmentation did not occur. This was observed for both static and flow conditions, Figure 4e and f.

## Discussion

In this work, we investigated the effect of simvastatin on an inflamed endothelium. We show that ECs treated with simvastatin or stimulated with TNF-α are still able to sense and respond to WSS. We quantified the influence that 12.5 dynes/cm^2^ of steady WSS, statin treatment, and TNF-α stimulation has on HAAEC morphology within three-dimensional tissue culture models. As in accordance with other published literature [40, 42, 44], we have observed an elongation of cells, Figure 3, and a significant decrease in EC SI, Figure 2, with the presence of a physiologically relevant WSS [29] of 12.5 dynes/cm^2^ when compared to the static control. We observed this trend for all of the conditions considered: with and without statin treatment, with or without mevalonate treatment, and with or without TNF-α stimulation.

It is well established that in the presence of WSS, ECs will elongate and align in the direction of flow [18, 40, 42, 44]. The elongation observed when ECs are exposed to steady WSS [45, 46] is believed to be a result of increased RhoA activity, which is known to be involved in cytoskeletal remodeling [28, 34]. The presence of an intact, elongated F-actin cytoskeleton, aligned in the direction of flow in response to WSS has been previously documented [42, 43].

We have also observed that EC stimulation with TNF-α causes a significant elongation (reduction in SI) visually and quantitatively under both static and flow conditions, Figures 2 and 3. There is a significant difference in SI observed between stimulated ECs with the addition of flow, which suggests that the cells are still responding to WSS despite the changes caused by stimulation. An interesting observation in this work is that both WSS and TNF-α stimulation have similar impacts on EC SI, Figure 2. There is no significant difference in the SI between non-stimulated ECs exposed to flow and static TNF-α stimulated ECs. This is true for each condition considered: control, simvastatin, and simvastatin and mevalonate. This suggests that the effect of WSS and TNF-α stimulation on RhoA activation is similar in magnitude.

The observed elongation is likely related to the EC cytoskeleton. The EC cytoskeleton is believed to mechanotransduce physical stimuli through the cell to biochemical changes [28, 47]. Research has shown that TNF-α stimulation causes the F-actin cytoskeleton to elongate and form dense actin bands [37, 48]. Additionally, TNF-α stimulation has also been shown to increase RhoA expression in ECs [37, 48] and other cell types [49], leading to the same effects as for the WSS-dependent RhoA activation. These effects appear additive, in that we have observed a significant decrease in EC SI between ECs exposed to 12.5 dynes/cm^2^ of steady WSS and TNF-α stimulated ECs exposed to 12.5 dynes/cm^2^ of steady WSS, Figure 2. This finding is consistent with that of Stroka et al. [50] who reported an increase in EC aspect ratio as well as elongation and alignment of the F-actin cytoskeleton with TNF-α stimulation. We also compared how the morphology of simvastatin treated ECs differed with TNF-α stimulation under both static and flow conditions, Figure 3. We observed that there is a significant increase in EC SI when TNF-α stimulated ECs are treated with 10 μM simvastatin, under both static and flow conditions, when compared to the stimulated control, Figure 2. We have also observed a significant decrease in EC SI between the non-stimulated and stimulated simvastatin treated ECs under static and flow conditions, Figure 2. This implies that the TNF-α stimulation still has an effect on simvastatin treated ECs.

It is believed that the activation of RhoA, by either steady WSS or stimulation with TNF-α, causes the F-actin cytoskeleton to become elongated and stretches out the cell [48]. Studies have shown that there is a link between statins and RhoA through the cholesterol biosynthesis pathway [10, 51], with statin treatment lowering RhoA activity [20, 52]. Statins work by blocking the rate-limiting step in the cholesterol biosynthesis pathway—the conversion of HMG-CoA to mevalonate. This prevents the formation of cholesterol, but also influences many intermediates within the pathway. Two important isoprenoid molecules are formed within this pathway, GGPP and FPP [10, 15, 51, 53], which serve as lipid attachments for small GTPases, such as Rho and Rac, which require posttranslational modifications [15, 54]. A decrease in FPP and GGPP will decrease the amount of active RhoA [12, 48, 55]. Ultimately, this decrease in protein expression is believed to lead to statin-induced F-actin cytoskeleton fragmentation and disorganization shown in both ECs [25] and stromal cells [52], leading to a round cellular morphology.

In this work, we have observed a decrease in EC SI with TNF-α stimulation, but an increase in EC SI with statin treatment, even with stimulation and WSS, Figure 2. Morphology has generally been correlated to cellular health. Researchers have found that in atheroprone regions of the vasculature, such as around bifurcations or curvature where there may be disturbed flow, ECs appear to have a cobblestone morphology [31, 42]. In contrast, ECs found in atheroprotective regions of steady flow appear elongated and have an atheroprotective genotype [18, 42, 56, 57]. This has led to the assumption that healthy ECs are elongated and unhealthy ECs are rounded and cobblestone. We have observed that TNF-α stimulation and WSS promote what is typically considered to be a healthy, atheroprotective EC morphology. We have also observed that statins influence the ECs to become rounder, which is typically indicative of an atheroprone morphology. This is counter-intuitive, as statins are thought to be beneficial to ECs and promote an atheroprotective genotype, and TNF-α stimulation is thought to promote an atheroprone genotype as it is commonly used as an inflammatory cytokine to simulate an atheroprone EC phenotype, such as for adhesion experiments [36]. Protein expression also indicates that TNF-α stimulation of ECs leads to an inflammatory phenotype as atheroprone markers, such as ICAM-1 and VCAM-1, are significantly increased [23, 24, 58]. We observed that the statin induced fragmentation of the EC F-actin cytoskeleton was not abrogated by either WSS or TNF-α stimulation on their own, although we believe them to both function through the cholesterol biosynthesis pathway.

In our work, we use mevalonate to ensure that the effects that we are observing due to statin treatment are regulated within the cholesterol biosynthesis pathway [59]. Indeed, we observe that there is no significant difference between the control condition and the simvastatin and mevalonate condition for both EC morphology, Figures 2 and 3, and F-actin cytoskeletal arrangement, Figure 4. This is true for both static and flow ECs, in accordance with previous results from our lab [22–25] and other literature sources [59–61], as well as non-stimulated and stimulated ECs. Mevalonate alone had no significant effect on cell proliferation, shape or F-actin organization (data not shown).

We have shown that both statin therapy and TNF-α stimulation induce changes in the cell, but that ECs still respond to WSS despite the statin-induced fragmentation. These results suggest that morphology and the state of the F-actin cytoskeleton may not be a true indicator of cellular health, and alternative methods of analysis should be considered. It also shows that EC mechanotransduction occurs despite changes in the F-actin cytoskeleton. Future work will focus on making a quantitative link between statin drugs and RhoA regulation, with the hopes of designing more specific and improved statin drugs.

## Conclusion

In this work, we studied how an inflamed (TNF-α stimulated) endothelium responds to steady WSS when treated with 10 μM of simvastatin. Statin treatment or TNF-α stimulation did not hinder the ability for ECs to sense and respond to WSS despite significant alterations in the F-actin cytoskeletal structure. Although statin drugs are thought to be beneficial to ECs, they caused the cells to adapt a rounded morphology and fragmented F-actin cytoskeleton, usually considered unhealthy. Conversely, TNF-α stimulation caused the cells to become more elongated, which is usually indicative of a healthy, atheroprotective EC. Neither WSS nor TNF-α stimulation was able to abrogate the statin-induced rounding of cells, or the F-actin cytoskeleton rearrangement due to statin treatment. ECs were still able to respond to WSS by elongating despite changes in the F-actin cytoskeleton structure. This work suggests that an alternate method of determining cellular health is necessary, as cellular morphology does not always correlate with cellular phenotype.

## Abbreviations

ECs: endothelial cells
TNF-α: tumor necrosis factor alpha
HMG-CoA: 3-hydroxy-3-methylglutaryl coenzyme A
WSS: wall shear stress
SI: shape index
